# Role of (-)-epigallocatechin-3-gallate in the osteogenic differentiation of human bone marrow mesenchymal stem cells: An enhancer or an inducer?

**DOI:** 10.3892/etm.2021.9725

**Published:** 2021-01-27

**Authors:** Pan Jin, Muyan Li, Guojie Xu, Kun Zhang, Li Zheng, Jinmin Zhao

Exp Ther Med 10:828–834, 2015; DOI: 10.3892/etm.2015.2579

Subsequently to the publication of the above paper, the authors were alerted to the fact that, in Fig. 4, the data panel for Fig. 4H partially overlapped with the data shown for Fig. 4E, such that these data panels were erroneously derived from the same original source. After having referred back to the original data, the authors have realized that the data panel for Fig. 4H was selected incorrectly for this Figure.

The corrected version of Fig. 4 in shown opposite, featuring the correct data for Fig. 4H. Note that this error did not seriously affect the overall conclusions reported in the study. The authors are grateful to the Editor for granting them the opportunity to publish this Corrigendum, and apologize to the readership of the Journal for any inconvenience caused.

## Figures and Tables

**Figure 4:**
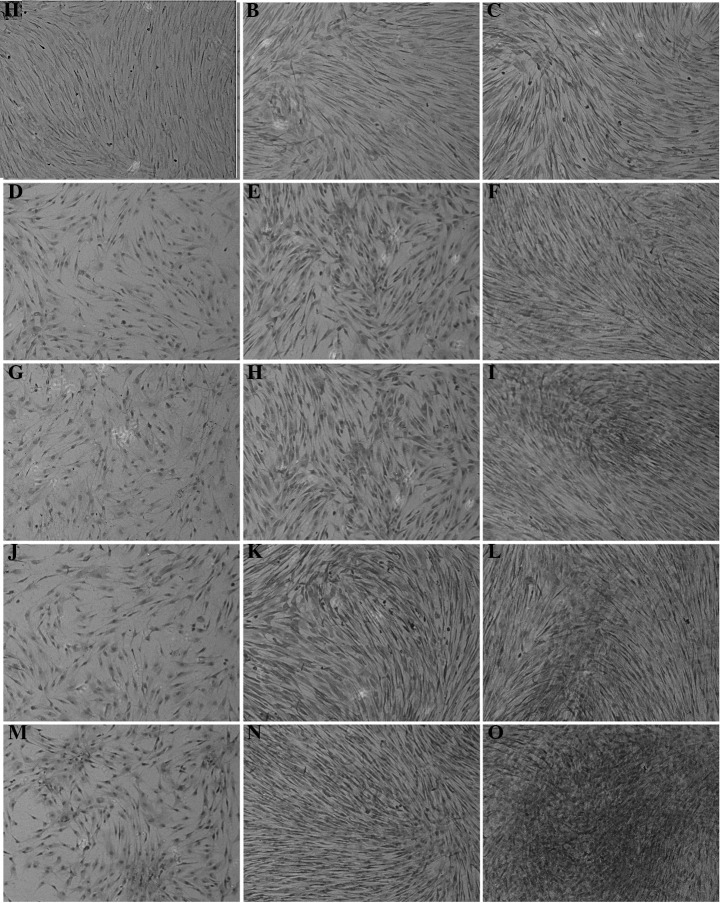
Immunohistochemical staining of bone morphogenetic protein 2 expression in human bone marrow mesenchymal stem cells (hBMSCs) of the three groups [negative control, hBMSCs cultured with pure culture medium; experimental, cells treated with culture medium containing 2.5, 5 and 10 M *μ*(-)-epigallocatechin-3-gallate (EGCG); positive control, cells cultured with osteogenesis-induced culture medium]. (A-C) Staining of hBMSCs in the negative control group on days 7, 14 and 21, respectively. (D-F) Staining of hBMSCs in the 2.5 *μ*M EGCG experimental group on days 7, 14 and 21, respectively. (G-I) Staining of hBMSCs in the 5 *μ*M EGCG experimental group on days 7, 14 and 21, respectively. (J-L) Staining of hBMSCs in the 10 *μ*M EGCG experimental group on days 7, 14 and 21, respectively. (M-O) Staining of hBMSCs in the positive control group on days 7, 14 and 21, respectively. The staining in the positive control group was evidently greater, while that in the other groups was dose-dependently upregulated.Magnification, x100.

